# 2-(2,5-Di­meth­oxy­phen­yl)-4,5-diphenyl-1-(prop-2-en-1-yl)-1*H*-imidazole

**DOI:** 10.1107/S1600536813015936

**Published:** 2013-06-15

**Authors:** Mehmet Akkurt, Shaaban K. Mohamed, Adel A. Marzouk, Antar A. Abdelhamid, Francisco Santoyo-Gonzalez

**Affiliations:** a Department of Physics, Faculty of Sciences, Erciyes University, 38039 Kayseri, Turkey; b Chemistry and Environmental Division, Manchester Metropolitan University, Manchester, M1 5GD, England; c Chemistry Department, Faculty of Science, Mini University, 61519 El-Minia, Egypt; d Pharmaceutical Chemistry Department, Faculty of Pharmacy, Al Azhar University, Egypt; e Mamedaliev Institute of Petrochemical Processes, National Academy of Sciences of Azerbaijan, Baku, Azerbaijan; f Chemistry Department, Faculty of Science, Sohag University, 82524 Sohag, Egypt; g Department of Organic Chemistry, Faculty of Science, Institute of Biotechnology, Granada University, Granada, E-18071, Spain

## Abstract

In the title compound, C_26_H_24_N_2_O_2_, the two phenyl and the 2,5-di­meth­oxy­phen­yl rings are inclined to the imidazole ring at dihedral angles of 30.38 (8), 56.59 (9) and 73.11 (9)°, respectively. In the crystal, mol­ecules are linked by pairs of C—H⋯O inter­actions into centrosymmetric dimers with graph-set notation *R*_2_^2^(8). C—H⋯π inter­actions are also observed.

## Related literature

For chemical properties and applications of imidazoles with an unsaturated side chain, see, for example: Koszykowska *et al.* (2009 ▶); Berezin *et al.* (2009 ▶); Rambo *et al.* (2010 ▶); Min *et al.* (2006 ▶). For similar structures, see: Akkurt *et al.* (2013*a* ▶,*b* ▶); Mohamed *et al.* (2013*a* ▶,*b* ▶). For hydrogen-bond motifs, see: Bernstein *et al.* (1995 ▶).
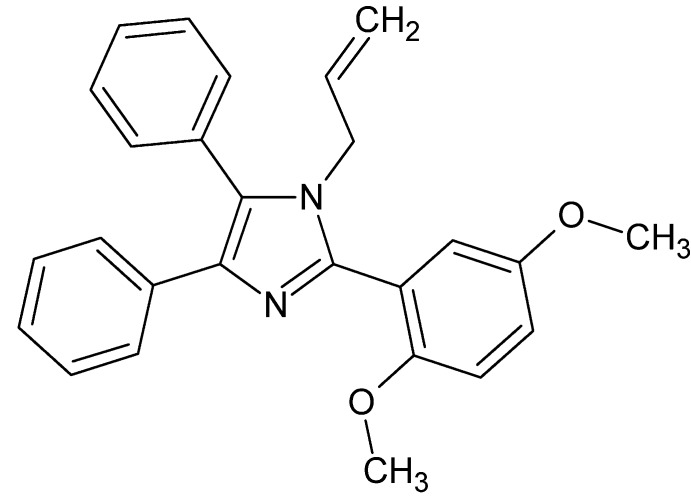


## Experimental

### 

#### Crystal data

C_26_H_24_N_2_O_2_
*M* *_r_* = 396.47
Triclinic, 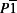

*a* = 8.3117 (14) Å
*b* = 10.5217 (17) Å
*c* = 13.425 (2) Å
α = 105.938 (2)°
β = 101.846 (2)°
γ = 107.772 (2)°
*V* = 1020.1 (3) Å^3^
*Z* = 2
Mo *K*α radiation
μ = 0.08 mm^−1^
*T* = 100 K
0.26 × 0.16 × 0.08 mm

#### Data collection

Bruker SMART APEX CCD area-detector diffractometer
Absorption correction: multi-scan (*SADABS*; Sheldrick, 2004 ▶) *T*_min_ = 0.979, *T*_max_ = 0.993
11527 measured reflections
4193 independent reflections
3184 reflections with *I* > 2σ(*I*)
*R*_int_ = 0.036

#### Refinement

*R*[*F* ^2^ > 2σ(*F* ^2^)] = 0.047
*wR*(*F* ^2^) = 0.125
*S* = 1.05
4193 reflections
273 parameters
H-atom parameters constrained
Δρ_max_ = 0.23 e Å^−3^
Δρ_min_ = −0.26 e Å^−3^

### 

Data collection: *SMART* (Bruker, 2001 ▶); cell refinement: *SAINT* (Bruker, 2001 ▶); data reduction: *SAINT*; program(s) used to solve structure: *SHELXS97* (Sheldrick, 2008 ▶); program(s) used to refine structure: *SHELXL97* (Sheldrick, 2008 ▶); molecular graphics: *ORTEP-3 for Windows* (Farrugia, 2012 ▶); software used to prepare material for publication: *WinGX* (Farrugia, 2012 ▶) and *PLATON* (Spek, 2009 ▶).

## Supplementary Material

Crystal structure: contains datablock(s) global, I. DOI: 10.1107/S1600536813015936/bx2444sup1.cif

Structure factors: contains datablock(s) I. DOI: 10.1107/S1600536813015936/bx2444Isup2.hkl

Supplementary material file. DOI: 10.1107/S1600536813015936/bx2444Isup3.cml

Additional supplementary materials:  crystallographic information; 3D view; checkCIF report

## Figures and Tables

**Table 1:** Hydrogen-bond geometry (Å, °) *Cg*1, *Cg*2 and *Cg*4 are the centroids of the N1/N2/C1–C3, C4–C9 and C19–C24 rings, respectively.

*D*—H⋯*A*	*D*—H	H⋯*A*	*D*⋯*A*	*D*—H⋯*A*
C20—H20⋯O1^i^	0.95	2.54	3.354 (2)	143
C14—H14⋯*Cg*2^ii^	0.95	2.63	3.4083 (19)	139
C25—H25*B*⋯*Cg*1^iii^	0.98	2.84	3.6337 (19)	139
C26—H26*C*⋯*Cg*4^iv^	0.98	2.95	3.908 (2)	166

Symmetry codes: (i) 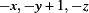; (ii) 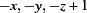; (iii) 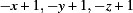; (iv) 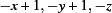.

**Fig. 1.**
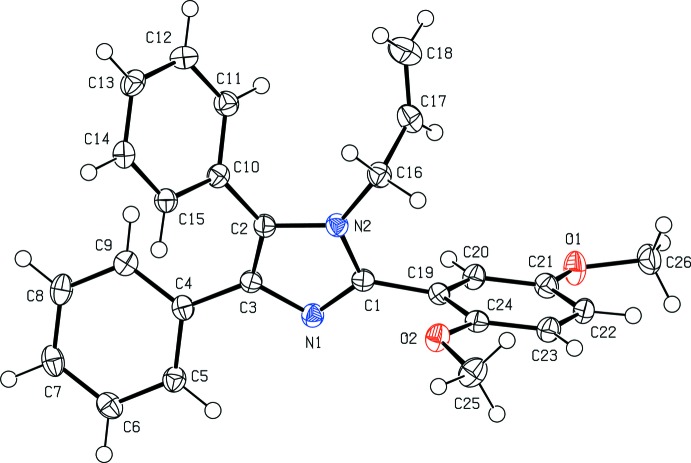
The molecular strcuture of the title compound with the atom numbering scheme. Displacement ellipsoids for non-H atoms are drawn at the 50% probability level.

**Fig. 2.**
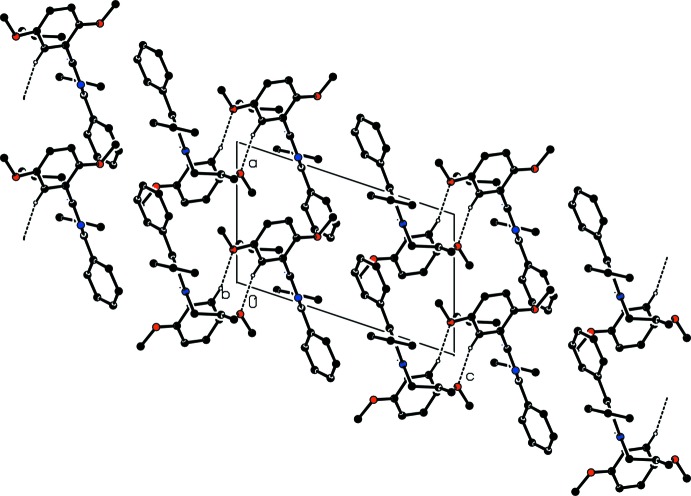
The hydrogen bonding and packing of the title compound viewing along the b axis. H atoms not involved in hydrogen bonds have been omitted for clarity.

## supplementary crystallographic information

### Comment

Recently, much attention has been devoted to vinyl and allyl N-substituted imidazole compunds due to their interesting properties and high reactivities. Such compounds in addition to threir flourecent properties (Berezin *et al*., 2009; Rambo *et al.*, 2010) they can polymerize to obtain chromophoric polymers (Koszykowska *et al.*, 2009). In addition their quaternery salts are acting as ionic catalists (Min *et al.*, 2006) which are widely used in green chemistry applications. In this context the title compound has been synthesized among series of allyl imidazole derivatives and herein we report its crystal structure.

In the title compound (I, Fig. 1), the two phenyl (C4–C9 and C10–C15) and 2-(2,5-dimethoxyphenyl) (C19–C24) rings are inclined to the N1/N2/C1–C3 imidazole ring at angles of 30.38 (8), 56.59 (9) and 73.11 (9)°, respectively. All bond lengths and angles are normal and are corresponding to those reported in a similar structure (Akkurt *et al.*, 2013*a*,*b*; Mohamed *et al.*, 2013*a*,*b*). In the crystal the molecules are linked by C— H··· O interactions into centrosymmetric dimers with graph-set notation R_2_^2^(8) (Bernstein *et al.*, 1995).C—H···π interactions are also observed,Table 1, Fig2.

### Experimental

The title compound was synthesized according to our reported method (Mohamed *et al.* 2013*a*) in 85% yield. Colourless prisms suitable for X-ray analyses were obtained by slow evaporation of a solution of (I) in ethanol, m.p. 471–473 K.

### Refinement

All H atoms were placed in geometrically, with C—H = 0.95–0.99 Å, and refined as riding with *U*_iso_(H) = 1.2 or 1.5*U*_eq_(C).

### Crystal data

**Table:**

C_26_H_24_N_2_O_2_	*Z* = 2
*M**_r_* = 396.47	*F*(000) = 420
Triclinic, *P*1	*D*_x_ = 1.291 Mg m^−^^3^
Hall symbol: -P 1	Mo *K*α radiation, λ = 0.71073 Å
*a* = 8.3117 (14) Å	Cell parameters from 2470 reflections
*b* = 10.5217 (17) Å	θ = 2.2–26.3°
*c* = 13.425 (2) Å	µ = 0.08 mm^−^^1^
α = 105.938 (2)°	*T* = 100 K
β = 101.846 (2)°	Prism, colourless
γ = 107.772 (2)°	0.26 × 0.16 × 0.08 mm
*V* = 1020.1 (3) Å^3^	

### Data collection

**Table:**

Bruker SMART APEX CCD area-detector diffractometer	4193 independent reflections
Radiation source: sealed tube	3184 reflections with *I* > 2σ(*I*)
Graphite monochromator	*R*_int_ = 0.036
phi and ω scans	θ_max_ = 26.5°, θ_min_ = 1.7°
Absorption correction: multi-scan (*SADABS*; Sheldrick, 2004)	*h* = −10→10
*T*_min_ = 0.979, *T*_max_ = 0.993	*k* = −13→13
11527 measured reflections	*l* = −16→16

### Refinement

**Table:**

Refinement on *F*^2^	Primary atom site location: structure-invariant direct methods
Least-squares matrix: full	Secondary atom site location: difference Fourier map
*R*[*F*^2^ > 2σ(*F*^2^)] = 0.047	Hydrogen site location: inferred from neighbouring sites
*wR*(*F*^2^) = 0.125	H-atom parameters constrained
*S* = 1.05	*w* = 1/[σ^2^(*F*_o_^2^) + (0.0613*P*)^2^ + 0.1427*P*] where *P* = (*F*_o_^2^ + 2*F*_c_^2^)/3
4193 reflections	(Δ/σ)_max_ < 0.001
273 parameters	Δρ_max_ = 0.23 e Å^−^^3^
0 restraints	Δρ_min_ = −0.26 e Å^−^^3^

### Special details

**Table:**

Geometry. Bond distances, angles *etc*. have been calculated using the rounded fractional coordinates. All su's are estimated from the variances of the (full) variance-covariance matrix. The cell e.s.d.'s are taken into account in the estimation of distances, angles and torsion angles
Refinement. Refinement on *F*^2^ for ALL reflections except those flagged by the user for potential systematic errors. Weighted *R*-factors *wR* and all goodnesses of fit *S* are based on *F*^2^, conventional *R*-factors *R* are based on *F*, with *F* set to zero for negative *F*^2^. The observed criterion of *F*^2^ > σ(*F*^2^) is used only for calculating -*R*-factor-obs *etc*. and is not relevant to the choice of reflections for refinement. *R*-factors based on *F*^2^ are statistically about twice as large as those based on *F*, and *R*-factors based on ALL data will be even larger.

### Fractional atomic coordinates and isotropic or equivalent isotropic displacement parameters (Å^2^)

**Table:**

	*x*	*y*	*z*	*U*_iso_*/*U*_eq_	
O1	0.22127 (15)	0.57663 (13)	−0.01565 (9)	0.0294 (4)	
O2	0.51365 (14)	0.51988 (12)	0.37054 (9)	0.0246 (3)	
N1	0.03320 (16)	0.34760 (14)	0.28257 (10)	0.0200 (4)	
N2	0.19265 (16)	0.21885 (14)	0.23560 (10)	0.0197 (4)	
C1	0.1675 (2)	0.34409 (16)	0.24524 (12)	0.0187 (4)	
C2	0.0647 (2)	0.13719 (17)	0.26932 (12)	0.0191 (5)	
C3	−0.0325 (2)	0.21832 (16)	0.29722 (12)	0.0187 (5)	
C4	−0.1839 (2)	0.18601 (17)	0.34015 (12)	0.0196 (5)	
C5	−0.2095 (2)	0.29766 (17)	0.41145 (13)	0.0221 (5)	
C6	−0.3463 (2)	0.26861 (19)	0.45693 (13)	0.0244 (5)	
C7	−0.4602 (2)	0.12872 (19)	0.43134 (14)	0.0257 (5)	
C8	−0.4395 (2)	0.01733 (19)	0.35825 (14)	0.0253 (5)	
C9	−0.3029 (2)	0.04563 (17)	0.31312 (13)	0.0219 (5)	
C10	0.05668 (19)	−0.00280 (17)	0.27544 (13)	0.0194 (4)	
C11	0.0409 (2)	−0.11491 (17)	0.18480 (13)	0.0234 (5)	
C12	0.0405 (2)	−0.24371 (18)	0.19320 (14)	0.0264 (5)	
C13	0.0564 (2)	−0.26260 (18)	0.29232 (14)	0.0248 (5)	
C14	0.0700 (2)	−0.15270 (17)	0.38256 (13)	0.0228 (5)	
C15	0.0696 (2)	−0.02425 (17)	0.37420 (13)	0.0210 (5)	
C16	0.3286 (2)	0.18032 (18)	0.19444 (13)	0.0229 (5)	
C17	0.2732 (2)	0.12340 (19)	0.07169 (14)	0.0280 (5)	
C18	0.2775 (3)	0.0033 (2)	0.01278 (16)	0.0392 (7)	
C19	0.2772 (2)	0.45508 (16)	0.21242 (13)	0.0199 (5)	
C20	0.2057 (2)	0.47090 (17)	0.11627 (13)	0.0221 (5)	
C21	0.3087 (2)	0.56926 (17)	0.08016 (13)	0.0220 (5)	
C22	0.4852 (2)	0.65115 (17)	0.14076 (14)	0.0238 (5)	
C23	0.5580 (2)	0.63844 (17)	0.23932 (13)	0.0233 (5)	
C24	0.4555 (2)	0.54150 (16)	0.27547 (13)	0.0202 (5)	
C25	0.6880 (2)	0.61529 (18)	0.44233 (14)	0.0292 (5)	
C26	0.3278 (2)	0.64720 (19)	−0.07022 (15)	0.0303 (6)	
H5	−0.13280	0.39400	0.42890	0.0270*	
H6	−0.36170	0.34510	0.50580	0.0290*	
H7	−0.55200	0.10910	0.46370	0.0310*	
H8	−0.51920	−0.07860	0.33910	0.0300*	
H9	−0.28980	−0.03130	0.26320	0.0260*	
H11	0.03020	−0.10310	0.11640	0.0280*	
H12	0.02920	−0.31920	0.13060	0.0320*	
H13	0.05790	−0.35020	0.29830	0.0300*	
H14	0.07970	−0.16520	0.45070	0.0270*	
H15	0.07820	0.05010	0.43670	0.0250*	
H16A	0.35140	0.10690	0.22150	0.0270*	
H16B	0.44100	0.26590	0.22340	0.0270*	
H17	0.23240	0.17810	0.03450	0.0340*	
H18A	0.31760	−0.05390	0.04740	0.0470*	
H18B	0.24060	−0.02650	−0.06460	0.0470*	
H20	0.08470	0.41400	0.07420	0.0270*	
H22	0.55700	0.71610	0.11540	0.0290*	
H23	0.67860	0.69670	0.28170	0.0280*	
H25A	0.77680	0.59950	0.40780	0.0440*	
H25B	0.70890	0.59740	0.51080	0.0440*	
H25C	0.69790	0.71440	0.45760	0.0440*	
H26A	0.39130	0.74890	−0.02480	0.0450*	
H26B	0.25120	0.63740	−0.14020	0.0450*	
H26C	0.41400	0.60380	−0.08310	0.0450*	

### Atomic displacement parameters (Å^2^)

**Table:**

	*U*^11^	*U*^22^	*U*^33^	*U*^12^	*U*^13^	*U*^23^
O1	0.0266 (6)	0.0360 (7)	0.0281 (7)	0.0075 (5)	0.0080 (5)	0.0209 (6)
O2	0.0216 (6)	0.0252 (6)	0.0223 (6)	0.0044 (5)	0.0028 (5)	0.0098 (5)
N1	0.0190 (7)	0.0203 (7)	0.0193 (7)	0.0059 (6)	0.0055 (6)	0.0074 (6)
N2	0.0188 (7)	0.0205 (7)	0.0210 (7)	0.0079 (6)	0.0073 (6)	0.0080 (6)
C1	0.0183 (7)	0.0192 (8)	0.0169 (8)	0.0064 (6)	0.0042 (6)	0.0059 (7)
C2	0.0181 (8)	0.0204 (8)	0.0156 (8)	0.0047 (7)	0.0040 (6)	0.0060 (7)
C3	0.0189 (8)	0.0187 (8)	0.0167 (8)	0.0058 (6)	0.0046 (6)	0.0062 (6)
C4	0.0189 (8)	0.0233 (9)	0.0193 (8)	0.0092 (7)	0.0060 (6)	0.0106 (7)
C5	0.0215 (8)	0.0219 (9)	0.0253 (9)	0.0090 (7)	0.0080 (7)	0.0108 (7)
C6	0.0245 (8)	0.0308 (9)	0.0249 (9)	0.0160 (8)	0.0102 (7)	0.0125 (8)
C7	0.0202 (8)	0.0357 (10)	0.0300 (9)	0.0133 (8)	0.0119 (7)	0.0189 (8)
C8	0.0177 (8)	0.0282 (9)	0.0298 (9)	0.0054 (7)	0.0051 (7)	0.0159 (8)
C9	0.0202 (8)	0.0221 (9)	0.0226 (9)	0.0078 (7)	0.0050 (7)	0.0086 (7)
C10	0.0152 (7)	0.0200 (8)	0.0215 (8)	0.0058 (6)	0.0044 (6)	0.0073 (7)
C11	0.0237 (8)	0.0248 (9)	0.0213 (9)	0.0090 (7)	0.0068 (7)	0.0085 (7)
C12	0.0296 (9)	0.0217 (9)	0.0248 (9)	0.0100 (7)	0.0080 (7)	0.0043 (7)
C13	0.0241 (8)	0.0206 (9)	0.0326 (10)	0.0099 (7)	0.0093 (7)	0.0123 (8)
C14	0.0205 (8)	0.0256 (9)	0.0236 (9)	0.0073 (7)	0.0077 (7)	0.0121 (7)
C15	0.0180 (8)	0.0216 (9)	0.0226 (8)	0.0068 (7)	0.0069 (7)	0.0072 (7)
C16	0.0194 (8)	0.0249 (9)	0.0260 (9)	0.0093 (7)	0.0084 (7)	0.0096 (7)
C17	0.0249 (9)	0.0345 (10)	0.0263 (9)	0.0113 (8)	0.0113 (7)	0.0114 (8)
C18	0.0452 (12)	0.0426 (12)	0.0293 (10)	0.0187 (10)	0.0155 (9)	0.0073 (9)
C19	0.0194 (8)	0.0190 (8)	0.0224 (8)	0.0073 (7)	0.0100 (7)	0.0067 (7)
C20	0.0197 (8)	0.0213 (8)	0.0234 (9)	0.0056 (7)	0.0057 (7)	0.0088 (7)
C21	0.0243 (8)	0.0246 (9)	0.0202 (8)	0.0111 (7)	0.0073 (7)	0.0104 (7)
C22	0.0253 (8)	0.0201 (8)	0.0293 (9)	0.0078 (7)	0.0132 (7)	0.0115 (7)
C23	0.0206 (8)	0.0222 (9)	0.0249 (9)	0.0062 (7)	0.0069 (7)	0.0077 (7)
C24	0.0214 (8)	0.0198 (8)	0.0201 (8)	0.0092 (7)	0.0070 (7)	0.0063 (7)
C25	0.0243 (9)	0.0263 (9)	0.0281 (9)	0.0045 (8)	−0.0003 (7)	0.0087 (8)
C26	0.0363 (10)	0.0328 (10)	0.0315 (10)	0.0149 (8)	0.0173 (8)	0.0193 (8)

### Geometric parameters (Å, º)

**Table:**

O1—C21	1.376 (2)	C20—C21	1.394 (3)
O1—C26	1.427 (2)	C21—C22	1.382 (2)
O2—C24	1.374 (2)	C22—C23	1.397 (2)
O2—C25	1.430 (2)	C23—C24	1.383 (2)
N1—C1	1.320 (2)	C5—H5	0.9500
N1—C3	1.387 (2)	C6—H6	0.9500
N2—C1	1.372 (2)	C7—H7	0.9500
N2—C2	1.388 (2)	C8—H8	0.9500
N2—C16	1.470 (2)	C9—H9	0.9500
C1—C19	1.479 (2)	C11—H11	0.9500
C2—C3	1.375 (2)	C12—H12	0.9500
C2—C10	1.480 (3)	C13—H13	0.9500
C3—C4	1.475 (2)	C14—H14	0.9500
C4—C5	1.400 (2)	C15—H15	0.9500
C4—C9	1.399 (3)	C16—H16A	0.9900
C5—C6	1.391 (3)	C16—H16B	0.9900
C6—C7	1.385 (3)	C17—H17	0.9500
C7—C8	1.388 (3)	C18—H18A	0.9500
C8—C9	1.386 (3)	C18—H18B	0.9500
C10—C11	1.393 (2)	C20—H20	0.9500
C10—C15	1.393 (2)	C22—H22	0.9500
C11—C12	1.390 (3)	C23—H23	0.9500
C12—C13	1.385 (3)	C25—H25A	0.9800
C13—C14	1.383 (2)	C25—H25B	0.9800
C14—C15	1.388 (3)	C25—H25C	0.9800
C16—C17	1.505 (2)	C26—H26A	0.9800
C17—C18	1.309 (3)	C26—H26B	0.9800
C19—C20	1.382 (2)	C26—H26C	0.9800
C19—C24	1.407 (2)		
			
C21—O1—C26	117.36 (14)	C5—C6—H6	120.00
C24—O2—C25	116.87 (14)	C7—C6—H6	120.00
C1—N1—C3	105.42 (14)	C6—C7—H7	120.00
C1—N2—C2	107.22 (14)	C8—C7—H7	120.00
C1—N2—C16	125.18 (15)	C7—C8—H8	120.00
C2—N2—C16	127.58 (15)	C9—C8—H8	120.00
N1—C1—N2	111.60 (15)	C4—C9—H9	120.00
N1—C1—C19	126.41 (16)	C8—C9—H9	120.00
N2—C1—C19	121.96 (15)	C10—C11—H11	120.00
N2—C2—C3	105.17 (15)	C12—C11—H11	120.00
N2—C2—C10	122.53 (15)	C11—C12—H12	120.00
C3—C2—C10	132.23 (16)	C13—C12—H12	120.00
N1—C3—C2	110.59 (15)	C12—C13—H13	120.00
N1—C3—C4	120.32 (15)	C14—C13—H13	120.00
C2—C3—C4	129.08 (16)	C13—C14—H14	120.00
C3—C4—C5	119.76 (16)	C15—C14—H14	120.00
C3—C4—C9	121.91 (15)	C10—C15—H15	120.00
C5—C4—C9	118.32 (16)	C14—C15—H15	120.00
C4—C5—C6	120.51 (17)	N2—C16—H16A	109.00
C5—C6—C7	120.36 (16)	N2—C16—H16B	109.00
C6—C7—C8	119.68 (17)	C17—C16—H16A	109.00
C7—C8—C9	120.17 (18)	C17—C16—H16B	109.00
C4—C9—C8	120.90 (16)	H16A—C16—H16B	108.00
C2—C10—C11	121.70 (15)	C16—C17—H17	118.00
C2—C10—C15	120.15 (15)	C18—C17—H17	118.00
C11—C10—C15	118.13 (16)	C17—C18—H18A	120.00
C10—C11—C12	120.84 (16)	C17—C18—H18B	120.00
C11—C12—C13	120.37 (16)	H18A—C18—H18B	120.00
C12—C13—C14	119.31 (17)	C19—C20—H20	120.00
C13—C14—C15	120.39 (16)	C21—C20—H20	120.00
C10—C15—C14	120.96 (15)	C21—C22—H22	120.00
N2—C16—C17	112.28 (14)	C23—C22—H22	120.00
C16—C17—C18	124.22 (18)	C22—C23—H23	120.00
C1—C19—C20	119.57 (15)	C24—C23—H23	120.00
C1—C19—C24	121.05 (15)	O2—C25—H25A	109.00
C20—C19—C24	119.34 (16)	O2—C25—H25B	109.00
C19—C20—C21	120.92 (16)	O2—C25—H25C	109.00
O1—C21—C20	115.32 (15)	H25A—C25—H25B	109.00
O1—C21—C22	125.10 (16)	H25A—C25—H25C	109.00
C20—C21—C22	119.58 (16)	H25B—C25—H25C	109.00
C21—C22—C23	120.05 (16)	O1—C26—H26A	109.00
C22—C23—C24	120.34 (16)	O1—C26—H26B	109.00
O2—C24—C19	115.12 (15)	O1—C26—H26C	109.00
O2—C24—C23	125.15 (15)	H26A—C26—H26B	109.00
C19—C24—C23	119.74 (15)	H26A—C26—H26C	109.00
C4—C5—H5	120.00	H26B—C26—H26C	109.00
C6—C5—H5	120.00		
			
C26—O1—C21—C22	15.8 (2)	C2—C3—C4—C5	148.45 (17)
C26—O1—C21—C20	−164.74 (15)	N1—C3—C4—C5	−29.8 (2)
C25—O2—C24—C19	−173.84 (15)	C3—C4—C5—C6	−176.81 (15)
C25—O2—C24—C23	6.6 (2)	C3—C4—C9—C8	177.09 (16)
C1—N1—C3—C2	0.58 (17)	C9—C4—C5—C6	2.2 (2)
C1—N1—C3—C4	179.16 (14)	C5—C4—C9—C8	−1.9 (2)
C3—N1—C1—N2	−0.36 (17)	C4—C5—C6—C7	−0.7 (3)
C3—N1—C1—C19	177.32 (15)	C5—C6—C7—C8	−1.3 (3)
C16—N2—C1—C19	0.9 (2)	C6—C7—C8—C9	1.6 (3)
C16—N2—C2—C10	4.5 (2)	C7—C8—C9—C4	0.0 (3)
C2—N2—C1—C19	−177.79 (14)	C2—C10—C11—C12	177.49 (17)
C16—N2—C2—C3	−178.34 (14)	C11—C10—C15—C14	1.2 (3)
C1—N2—C2—C10	−176.80 (14)	C2—C10—C15—C14	−177.22 (16)
C16—N2—C1—N1	178.73 (13)	C15—C10—C11—C12	−0.9 (3)
C2—N2—C16—C17	97.69 (19)	C10—C11—C12—C13	−0.2 (3)
C1—N2—C16—C17	−80.8 (2)	C11—C12—C13—C14	1.0 (3)
C2—N2—C1—N1	0.00 (17)	C12—C13—C14—C15	−0.7 (3)
C1—N2—C2—C3	0.34 (16)	C13—C14—C15—C10	−0.4 (3)
N1—C1—C19—C20	−72.9 (2)	N2—C16—C17—C18	−129.1 (2)
N1—C1—C19—C24	109.5 (2)	C1—C19—C24—C23	176.09 (16)
N2—C1—C19—C20	104.59 (19)	C20—C19—C24—O2	178.86 (15)
N2—C1—C19—C24	−73.1 (2)	C1—C19—C24—O2	−3.5 (2)
N2—C2—C10—C15	121.30 (18)	C24—C19—C20—C21	1.2 (3)
C3—C2—C10—C11	126.7 (2)	C20—C19—C24—C23	−1.6 (3)
C3—C2—C10—C15	−55.0 (3)	C1—C19—C20—C21	−176.52 (16)
N2—C2—C10—C11	−57.0 (2)	C19—C20—C21—C22	0.6 (3)
N2—C2—C3—C4	−178.98 (15)	C19—C20—C21—O1	−178.89 (16)
N2—C2—C3—N1	−0.57 (17)	C20—C21—C22—C23	−2.0 (3)
C10—C2—C3—N1	176.17 (16)	O1—C21—C22—C23	177.44 (16)
C10—C2—C3—C4	−2.3 (3)	C21—C22—C23—C24	1.6 (3)
C2—C3—C4—C9	−30.5 (3)	C22—C23—C24—C19	0.2 (3)
N1—C3—C4—C9	151.19 (15)	C22—C23—C24—O2	179.71 (16)

### Hydrogen-bond geometry (Å, º)

Cg1, Cg2 and Cg4 are the centroids of the N1/N2/C1–C3, C4–C9 and C19–C24 rings, respectively.

**Table:**

*D*—H···*A*	*D*—H	H···*A*	*D*···*A*	*D*—H···*A*
C20—H20···O1^i^	0.95	2.54	3.354 (2)	143
C14—H14···*Cg*2^ii^	0.95	2.63	3.4083 (19)	139
C25—H25*B*···*Cg*1^iii^	0.98	2.84	3.6337 (19)	139
C26—H26*C*···*Cg*4^iv^	0.98	2.95	3.908 (2)	166

Symmetry codes: (i) −*x*, −*y*+1, −*z*; (ii) −*x*, −*y*, −*z*+1; (iii) −*x*+1, −*y*+1, −*z*+1; (iv) −*x*+1, −*y*+1, −*z*.
